# Heat-Induced Hatching: Clarifying Effects of Hydration and Heating Rate on Behavioral Thermal Tolerance of Red-Eyed Treefrog Embryos

**DOI:** 10.1093/iob/obaf023

**Published:** 2025-06-05

**Authors:** Estefany Caroline Guevara-Molina, Fernando Ribeiro Gomes, Karen M Warkentin

**Affiliations:** Department of Physiology, Institute of Biosciences, Laboratory of Behavior and Evolutionary Physiology, University of São Paulo, São Paulo 05508-900, Brazil; Department of Physiology, Institute of Biosciences, Laboratory of Behavior and Evolutionary Physiology, University of São Paulo, São Paulo 05508-900, Brazil; Department of Biology, Boston University, Boston, MA 02215, USA; Smithsonian Tropical Research Institute, 0843-03092 Balboa, Panamá

## Abstract

Anurans are one of the most diverse groups of vertebrates but also most threatened by current climate change effects such as increasing environmental temperatures and more frequent and prolonged periods without rain. Many tropical anurans lay terrestrial eggs that are particularly vulnerable to drying and warming. In some such species, embryos hatch prematurely to escape from drying eggs. In red-eyed treefrogs, *Agalychnis callidryas*, embryos hatch early to escape both drying and excessive warming, expressing a behavioral thermal tolerance (i.e., VT_Max_). Prior research suggested that drying reduces the VT_Max_ of embryos. However, because hydrated clutches warmed more slowly, the effect of drying on VT_Max_ was confounded with that of faster warming. To disentangle these dynamics, we designed a novel apparatus to warm terrestrial frog egg-clutches at controlled rates and minimize evaporative cooling. We independently manipulated clutch hydration and heat input to assess their individual and combined effects on embryo VT_Max_. Proportional egg-volume loss was similar across hydration × heat input categories. High heat input resulted in higher clutch warming rates and shorter trial durations, across hydration levels. Within clutches, warming rates differed between back and surface thermocouple positions, generating thermal gradients as warming progressed. Clutch dehydration reduced embryo VT_Max_, with no main or interacting effect of heat input. This evidence that egg drying reduces thermal tolerance across testing conditions supports a direct role for hydration in the behavioral decisions of warming embryos, rather than an indirect effect mediated by changes in evaporative cooling. It suggests that embryos assessing risk integrate information about hydration, and perhaps changes in hydration, with information about current and changing temperature. These findings highlight the value of methods to independently manipulate hydration and heating rate, showing the complexity of thermal ecology in embryonic ectotherms. We encourage further research on temperature and hydration effects on embryo hatching to better understand tropical anurans’ adaptive strategies under climate change.

## Resumen

Los anuros constituyen uno de los grupos de vertebrados más diversos, pero también son los más amenazados por los efectos actuales del cambio climático, como el aumento de las temperaturas ambientales y la mayor frecuencia y prolongación de los periodos sin lluvia. Muchos anuros tropicales depositan huevos terrestres, que son particularmente vulnerables a la desecación y el calentamiento. En varias de estas especies, los embriones eclosionan prematuramente para evitar la desecación de los huevos. En la rana arbórea de ojos rojos, *Agalychnis callidryas*, los embriones eclosionan prematuramente para evitar tanto la desecación como el calentamiento excesivo, expresando una tolerancia térmica conductual (es decir, VT_Max_). Investigaciones previas sugirieron que la desecación redujo la VT_Max_ de los embriones. Sin embargo, dado que las puestas hidratadas se calentaron más lento, el efecto de la desecación sobre la VT_Max_ se confundió con el de un calentamiento más rápido. Para desentrañar esta dinámica, diseñamos un novedoso aparato para calentar las puestas de huevos de ranas terrestres a ritmos controlados y minimizar el enfriamiento por evaporación. Manipulamos de forma independiente la hidratación de la puesta y el aporte de calor para evaluar sus efectos individuales y combinados sobre la VT_Max_ del embrión. La pérdida proporcional del volumen del huevo fue similar en todas las categorías de hidratación × entrada de calor. Una entrada de calor alta resultó en mayores tasas de calentamiento de la nidada y menores duraciones de prueba, en todos los niveles de hidratación. Dentro de las nidadas, las tasas de calentamiento difirieron entre las posiciones del termopar posterior y superficial, generando gradientes térmicos a medida que avanzaba el calentamiento. La deshidratación de la nidada redujo el VT_Max_ de los embriones, sin un efecto principal ni interactivo de la entrada de calor. Esta evidencia de que el secado del huevo reduce la tolerancia térmica en las condiciones de prueba respalda un papel directo de la hidratación en las decisiones de comportamiento de los embriones en calentamiento, en lugar de un efecto indirecto mediado por cambios en el enfriamiento por evaporación. Esto sugiere que los embriones que evalúan el riesgo integran información sobre la hidratación, y posiblemente cambios en la hidratación, con información sobre la temperatura actual y cambiante. Estos hallazgos resaltan el valor de los métodos para manipular de forma independiente la hidratación y la tasa de calentamiento, mostrando la complejidad de la ecología térmica en los embriones de ectotermos. Fomentamos la investigación adicional sobre los efectos de la temperatura y la hidratación en la eclosión de embriones para comprender mejor las estrategias adaptativas de los anuros tropicales ante el cambio climático.

## Resumo

Os anuros constituem um dos grupos de vertebrados mais diversos, mas também são os mais ameaçados pelos efeitos atuais das mudanças climáticas, como o aumento das temperaturas ambientais e períodos mais frequentes e longos sem chuva. Muitos anuros tropicais depositam ovos terrestres, que são particularmente vulneráveis à dessecação e ao aquecimento. Em várias dessas espécies, os embriões eclodem prematuramente para evitar a dessecação dos ovos. Na perereca de olhos vermelhos, *Agalychnis callidryas*, os embriões eclodem prematuramente para evitar tanto a dessecação quanto o aquecimento excessivo, expressando uma tolerância térmica comportamental (ou seja, VT_Max_). Pesquisas previas sugeriram que a dessecação reduziu a VT_Max_ dos embriões. Entretanto, como as desovas hidratadas aqueceram mais lentamente, o efeito da dessecação na VT_Max_ foi confundido com o de um aquecimento mais rápido. Para desvendar essa dinâmica, desenhamos um novo dispositivo para aquecer desovas de pererecas terrestres em taxas controladas e minimizar o resfriamento evaporativo. Nós manipulamos independentemente a hidratação da desova e a entrada de calor para avaliar seus efeitos individuais e combinados na VT_Max_ embrionária. A perda proporcional do volume do ovo foi semelhante em todas as categorias de hidratação × entrada de calor. Alta entrada de calor resultou em maiores taxas de aquecimento da desova e durações mais curtas dos testes, em todos os níveis de hidratação. Dentro das desovas, as taxas de aquecimento diferiram entre as posições dos termopares posteriores e superficiais, gerando gradientes térmicos à medida que o aquecimento progredia. A desidratação da desova reduziu a VT_Max_ dos embriões, sem efeito principal ou interativo da entrada de calor. Essa evidência de que a secagem dos ovos reduz a tolerância térmica sob as condições de teste apoia um papel direto da hidratação nas decisões comportamentais de aquecimento dos embriões, em vez de um efeito indireto mediado por mudanças no resfriamento evaporativo. Isso sugere que os embriões que avaliam riscos integram informações sobre a hidratação, e possivelmente mudanças na hidratação, com informações sobre a temperatura atual e variável. Essas descobertas destacam o valor dos métodos para manipular de forma independente a hidratação e a taxa de aquecimento, mostrando a complexidade da ecologia térmica em embriões ectotérmicos. Incentivamos mais pesquisas sobre os efeitos da temperatura e da hidratação na eclosão de embriões para entender melhor as estratégias adaptativas dos anuros tropicais diante das mudanças climáticas.

## Introduction

Anurans are the most threatened group of vertebrates, and particularly at risk in the Neotropics, with climate change posing increasing danger and exacerbating other risks (e.g., interaction of increased temperature and drought with deforestation) (Luedtke et al. 2023; Wu et al. 2024). Across all life stages, anurans are highly dependent on specific thermal and hydric conditions for their development, growth, and survival. Harmful effects of heat and dehydration are well documented for Neotropical tadpoles, juveniles, and adults (e.g., O'Regan et al. 2014; Perotti et al. 2018; Guevara-Molina et al. 2020; Rudin-Bitterli et al. 2020; Greenberg and Palen 2021; Hoffmann et al. 2021; Sanabria et al. 2021; Delgado-Suazo and Burrowes 2022; Turriago et al. 2022; Cicchino et al. 2023; Maenaka et al. 2023). However, we know less about such effects on eggs. Most studies on early life stages have focused on dehydration risk, with little attention to excessive warming or the combined effects of heat and dryness (Salica et al. 2017; Rudin-Bitterli et al. 2020).

Many species of Neotropical anurans have terrestrial or semi-terrestrial development, laying eggs on terrestrial substrates, either hanging above water bodies (e.g., *Centrolenidae* and *Hylidae*) or in moist leaf litter (e.g., *Dendrobatidae*) or soil (e.g., *Craugastoridae*) (Crump 2015; Liedtke et al. 2022). These eggs are exposed to fluctuating air temperatures and irregular rainfall, which can lead to rapid warming and dehydration (Mueller et al. 2019; Vorsatz et al. 2022). The increasing frequency of short rainless periods during rainy seasons probably results in more egg death than occurs during extended rainless periods (e.g., dry seasons), when the frogs are not laying eggs (Touchon and Warkentin 2009). The thermal and hydric environment influence key physiological processes, including growth, development, metamorphosis, and oxygen exchange (Gómez-Mestre et al. 2010; Touchon and Warkentin 2011), impacting both developmental trajectories and mortality risks of embryos and larvae (Mitchell and Seymour 2000; Mueller et al. 2011; Arrighi et al. 2013). As well as mortality, dehydration also increases the risk of premature hatching in tropical frogs (Touchon et al. 2011; Salica et al. 2017; González et al. 2021). Thus, a combination of elevated temperatures and dry weather increases the vulnerability of embryos in terrestrial clutches (Guevara-Molina et al. 2022). Climate change is increasing the frequency of heat waves and short-term rainfall fluctuations, which are directly influencing the persistence and distribution of anuran populations (Blaustein et al. 2010; Catenazzi and Kupferberg 2017; Kissel et al. 2019). Understanding how embryos respond behaviorally and physiologically to these changes is key to assessing species' responses to current climate impacts (Vorsatz et al. 2022; Pottier et al. 2022). Measures of embryonic thermal tolerance in anurans could enable estimates of how frog embryos cope with external environmental conditions that increase the risk of reaching dangerous levels of temperature and dehydration *in ovo*.

Thermal tolerance in embryonic ectotherms is commonly assessed using metrics such as critical thermal limits (CT_Min_ and CT_Max_) and lethal temperatures (LT_50_), which estimate the effects of temperature on physiological function and survival (Angilletta et al. 2013; Smith et al. 2015; Turriago et al. 2015; Hall and Warner 2020; Hall and Sun 2021; Cowan et al. 2023). In embryos, these thresholds have been associated with cessation of movement (Cowan et al. 2023; Ern et al. 2023) and cardiovascular dysfunction (Angilletta et al. 2013; Hall and Sun 2021; Cowan et al. 2023). In addition, embryonic chronic heat tolerance, is estimated using LT_50_, the constant temperature resulting in 50% mortality and/or hatching failure (Hall and Sun 2021). Recently, we reported the first measurement of behavioral thermal tolerance of embryos, measured as voluntary thermal maximum (VT_Max_), in the red-eyed treefrog *Agalychnis callidryas*, based on the temperature at which embryos performed an escape-hatching behavior to leave their warming eggs (Guevara-Molina et al. 2022). This non-lethal metric focuses on animals’ behavioral avoidance responses to warming, that is, moving to a cooler place. It has been previously measured in anuran juveniles (Guevara-Molina et al. 2020) and adults (Diaz-Ricaurte et al. 2020). However, species that show environmentally cued hatching in response to egg-stage risk (Warkentin 2011a, 2011b) are candidates for measuring VT_Max_ in embryos (Guevara-Molina et al. 2022).

Understanding thermal tolerances requires integrating factors that influence both thermal exposure and the physiological responses to it (Ruthsatz et al. 2022, 2024). In terrestrial frog embryos, clutch hydration and heating rates—affected by weather and microhabitat—can shape thermal responses. For example, reduced rainfall during breeding season increases the risk of egg mortality (Allan and Soden 2008; Salica et al. 2017). Moreover, the highly gelatinous clutches of some species can absorb and retain substantial amounts of water during development, while clutches of other species contain very little jelly (Delia et al. 2020; Güell et al. 2024). Both clutch structure and hydration affect available thermal and hydric regulation mechanisms, such as evaporative cooling, and thereby the heating rates of clutches (Guevara-Molina et al. 2022). Clutches with more jelly, and in a better-hydrated state, have greater capacity for evaporative cooling. Embryos in such clutches may therefore cope better with environmental warming compared to embryos in less gelatinous or drier clutches (Guevara-Molina et al. 2022).

Thermal tolerance (e.g., CT_Max_) is influenced by acclimation, body size, hydration, heating rates, and habitat use across different life stages and taxa (Anderson and Andrade 2017; Agudelo-Cantero and Navas 2019; Illing et al. 2020; Delgado-Suazo and Burrowes 2022; Penman et al. 2023; Turriago et al. 2023; Ruthsatz et al. 2024). Nonetheless, behavior might also influence the relationships among these variables, and the effects of warming and hydration do not always combine as predicted by physics alone. For instance, in juvenile bullfrogs experiencing high heating rates, hydrated individuals warmed more quickly than dry ones, and this more rapid warming was associated with higher CT_Max_ but not with higher VT_Max_, that is, individual warming rates did not alter VT_Max_ (Guevara-Molina et al. 2020). The effects of body mass, developmental stage, acclimation, initial body temperatures, or other organism-specific variables have, however, not been explored for behavioral thermal tolerance (VT_Max_) of anurans, much less tadpoles or embryos. Thus, the physical properties of the organism and the environment that surrounds it, heat perception, and behavioral adjustments to heat are all relevant for understanding thermal tolerance limits. For embryos, studies of thermal tolerance rarely address potential modulating factors (e.g., heating rates, hydration levels), particularly in combination. This is necessary to enable extrapolation of experimental measurements to more realistic climate change scenarios for anurans with terrestrial oviposition. However, testing and disentangling the interacting effects of multiple variables that may directly or indirectly modulate embryonic thermal tolerance requires improved methods to manipulate those variables.

The red-eyed treefrog, *A. callidryas*, is a model species for understanding how embryonic behavior and early hatching are affected by environmental factors (Warkentin 1995, 2000, 2002; Salica et al. 2017; Warkentin et al. 2017). The embryo VT_Max_ was recently measured in both hydrated and dehydrated clutches, revealing that embryos exhibited lower heat tolerance in dry clutches than in wet ones (Guevara-Molina et al. 2022). This study also found that, under the initial standardized heat input method, clutch hydration level substantially altered the warming rate, largely due to differences in evaporative cooling. Because high hydration level, slow heating rate, and long duration of exposure were all confounded, it was unclear which variables directly affect embryo VT_Max_ and if, or how, they interact. Here, we directly tested how warming rates and hydration levels of egg clutches, both independently and in combination, affect the behavioral thermal tolerance of terrestrial anuran embryos, using *A. callidryas* as a model species. We developed and implemented a novel heating method that improves control of heating rates across hydration levels (Guevara-Molina et al. 2022), enabling clearer assessment of these effects. Based on the results from other species and life stages, we hypothesized that both heating rate and hydration would affect VT_Max_. Our controlled heating approach provides a replicable framework to assess embryonic thermal tolerance in other anuran species with terrestrial oviposition, particularly those that have rapid hatching responses to environmental threats. Understanding how thermal tolerance and heating dynamics interact may elucidate the mechanisms underlying frog embryos’ responses to climate change stressors.

## Materials and methods

This study was carried out at the Smithsonian Tropical Research Institute in Gamboa, Panama, during the rainy season (June–August) of 2022. To assess how hydration and/or heating rate affect the VT_Max_ of *A. callidryas* embryos, we collected 28 clutches on leaves from the Experimental Pond (9.120894 N, 79.704015 W; 45 m asl). All clutches were fully maternally hydrated during oviposition the night before, still in cleavage stages (i.e., before gastrulation; see Warkentin 2017), and egg sizes appeared similar. We divided these clutches into four experimental groups (7 replicates each) to test the effects of two hydration levels (wet and dry) and two heating rates (fast and slow) on embryo hatching. We maintained the clutches and conducted the experiments in an ambient temperature and humidity laboratory (Temperature: mean 26.19°C ± 0.63 SD, range = 24.9–28.1°C; Humidity: 90.36% ± 3.35 SD, range = 81–96%) with approval from the STRI Animal Care and Use Committee (2020–0318-2023-A1), and research permits from the Panamanian Ministry of the Environment (ARB-044–2021).

### Clutch setup and hydration treatments

We transported clutches on leaves to an ambient temperature laboratory. Then, to standardize the substrate, avoid thermal variation due to variation in leaves (Guevara-Molina et al. 2022), and improve thermal coupling to our heating apparatus, we immediately transferred each clutch to a Plexiglas card (6.7 cm width × 11 cm height). We manually peeled the clutch off its leaf and, carefully maintaining the original orientation and position of the eggs, placed it on the Plexiglass. We laid these Plexiglas-mounted clutches flat and left them unsprayed for at least 6 h to allow the jelly to adhere to the Plexiglas. We then placed each clutch in a near-vertical position in a plastic cup with dechlorinated water below the eggs and randomly assigned it to a wet or dry treatment. We used the same hydration protocol as Guevara-Molina et al. (2022). In the wet treatment, clutches were maintained in a plastic box with a partially screened lid (egg humidor) and hydrated by automatically spraying them with rainwater every 75 min for 15 s, using a MistKing system (https://junglehobbies.com/mistking). We checked clutches at least twice daily and removed excess water that accumulated in cups. In the dry treatment, clutches were placed in a similar screened box without automatic hydration. If visual inspection indicated that their egg size decreased to ≤4.0 mm diameter (Salica et al. 2017), we manually misted them for 5 s once daily.

### Controlled heating method

**Fig. 1.**
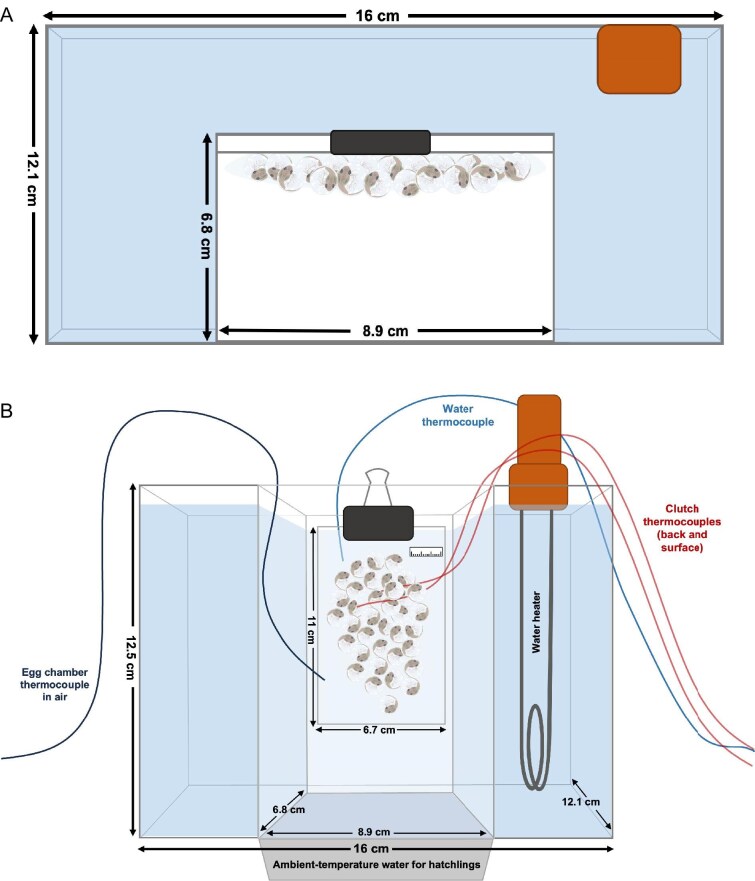
Illustration of our custom-designed U-shaped plexiglass aquarium for heating terrestrial clutches. (A) Top view of the apparatus indicating the air chamber where egg clutches were located. (B) Front view of the apparatus indicating the location of the thermocouples and the electric water heater. Under the apparatus, a container with dechlorinated water at room temperature was placed to catch hatched tadpoles. The egg chamber had an opening at the bottom so that when embryos hatched, they fell directly into the water. Not shown are the supports for the aquarium, placed on either side of the water for hatchlings, and the lid that covered the air chamber to maintain high humidity around eggs, leaving only a small opening for thermocouple wires.

To warm clutches at a controlled rate and minimize variation due to evaporative cooling, we designed a novel heating apparatus. This consisted of a U-shaped Plexiglas aquarium (16 cm length × 12.1 cm width × 12.5 cm height) with the open side of the U (8.9 cm length × 6.8 cm width × 12.5 cm height) sealed with a pane of glass to create an open-bottomed air-filled chamber (Fig. 1). For experiments, the chamber held an egg clutch in air, surrounded on three sides by water (Fig. 1). The water in the aquarium (1230 mL) was heated with an electric water heater (60 Hz, 2000 W; 4.5 cm width × 23 cm total length with metal part ≈ 11.5 cm long) that was positioned in a back corner of the aquarium, with the left or right corner randomized for each experiment. The Plexiglas cards supporting clutches (see above) were sized to fit in the chamber and provided a consistent, strong thermal coupling so that heating the water warmed the clutch from the back by conduction, through the 2 layers of Plexiglas (Fig. 1). We observed and video-recorded embryo responses through the glass at the front. With a dimmer connected to the heater, we established 2 levels of heat input (high and low) that generated fast and slow water heating rates (trial means ± SD: 0.39 ± 0.03 and 0.21 ± 0.01°C/min, respectively). We define heat input as a categorical treatment variable—either high or low—representing the heater setting. In contrast, continuous heating rate variables refer to rates of temperature increase (°C/min) measured by thermocouples placed at specific locations.

When clutches reached 4 days of age, we set them up to assess the embryos’ VT_Max_ (Guevara-Molina et al. 2022). First, we checked for undeveloped and/or less developed embryos and, if possible, removed them. Then, we placed 2 Omega T-Type Thermocouples in the clutch (Fig. 1B). At this age, embryos are hatching-competent but insensitive to low-amplitude vibrations and easy to handle without inducing hatching (Warkentin et al. 2022; Jung et al. 2025). To assess thermal gradients in the clutch during heating, we placed one thermocouple at the air-exposed surface and inserted the other through the jelly to contact the Plexiglas at the back of the clutch. We also placed a scale next to the clutch for egg size measurements. In addition, we placed one thermocouple in the water behind the eggs and another in the air inside the chamber to track the temperatures and heating rates of the water near the eggs and the air surrounding them. We mounted the instrumented clutch at the back of the air chamber in the aquarium. To maintain the initial hydration level overnight, we sprayed the clutch with a little rainwater if necessary. We placed the aquarium with the clutch above a tray of dechlorinated, ambient temperature water to catch hatched tadpoles (Fig. 1B) and covered the top with a lid to exclude insects and maintain high humidity in the air around eggs.

### Measurement of voluntary thermal maximum (VT_Max_)

The next day, when embryos were 5 days of age and midway through their plastic hatching period (age 3–7 days; see Warkentin et al. 2022), we conducted the heating experiment. We connected the thermocouples to a PicoLog USB TC-08 Data Logger and computer using the PicoLog Data logging software Version 6.2.8 to record the temperatures every 10 s. We used a Sony Handycam 4K (FDR-AX53) video camera with Zeiss Vario-Sonnar T* lens mounted on a tripod in front of the aquarium to record embryo behavior during heating, as well as the time each embryo hatched. Videos were recorded at 3840 × 2160-pixel resolution, 30 frames per second, using an H.264 video codec. We observed each experiment continuously and narrated observations of each hatching event, including the time and temperature of the nearest thermocouple (i.e., our estimate of individual VT_Max_), onto the audio track for later transcription. When the temperature at either of the 2 thermocouples in the clutch reached 42–42.5°C we stopped the experiment; in pilot work with this apparatus, we observed hatching at temperatures as high as 41°C (unlike Guevara-Molina et al. 2022, where experiments stopped at 40°C). To stop the experiment, we turned off the water heater then carefully removed the Plexiglas card with the clutch from the heating apparatus and placed it in a cup with water, at ambient temperature. After that, we checked all unhatched embryos for blood circulation, evident from the heartbeat and/or pulse in the external gills. We also attempted to induce hatching by jiggling eggs with a blunt probe, providing a standardized and highly effective mechanosensory hatching stimulus (Warkentin et al. 2017; Jung et al. 2020). If any embryos did not hatch, we left them for 24 h and then attempted to induce hatching by jiggling once more; any that did not respond were considered dead. We maintained all hatched tadpoles (i.e., both those that hatched during heating and jiggling) in the lab for 5 days, feeding them Sera Micron ad libitum and monitoring them for normal development and survival. Then, they were released in the Experimental Pond.

### Egg sizes and volume loss during trial

We measured a subsample of eggs in each clutch from initial and final still frames from the video recorded from each experiment. We used Image J (NIH) software (Schneider et al. 2012) to measure the diameters of 20 randomly selected eggs per clutch from the initial photo, or all visible eggs for 2 clutches with just 18 such eggs. From the final photo, we measured all remaining fully visible unhatched eggs (in all cases <20). We calculated initial and final egg volumes using the formula *V* = (4/3) π *r*^3^ (Guevara-Molina et al. 2022) and used those to calculate proportional egg-volume loss.

### Clutch heating rates

We estimated 2 trial-mean heating rates for each clutch as the difference between final and initial temperature, divided by trial duration, using data from the thermocouples located at back and surface positions within the clutch. We averaged these to estimate an overall clutch-mean heating rate. In addition, to examine variation in the heating rates of clutches over time, we estimated a series of heating rates averaged over 5-min periods for each location within each clutch (henceforth, 5-min heating rates). In most trials, water temperature behind the clutch and both clutch temperatures began increasing immediately upon turning on the water heater. However, in 5 trials, those 3 thermocouples recorded an initial period without warming. To base our analysis on our best estimate of embryo experience, we set the start time for warming trials based on when measured warming began, removing any initial “no warming” period (5 min for 3 clutches, and 15 and 20 min for one clutch each). All trial durations, hatching times, and initial temperatures reported are based on these start times for actual warming. At the start of trials, because temperatures were similar at both locations within a clutch (initial difference, mean ± SD: 0.13 ± 0.13°C, range: 0.01–0.71°C), we consider the thermocouple measurements to be good estimates of embryo temperatures throughout the clutch. During warming, thermal gradients developed within clutches (final difference: 1.72 ± 1.15°C, 0.21–5.12°C), thus individual embryo temperatures may have differed from either measured location. Particularly in clutches with large thermal gradients, and for embryos distant from both thermocouples, this limits the precision of their VT_Max_ estimates.

### Statistical analysis

Before comparing across treatments, we first tested if the data met parametric assumptions. If they did not, we employed non-parametric analyses. Accordingly, we used Mann–Whitney–Wilcoxon U tests to assess if initial egg size differed across hydration treatments and to compare mean water heating rate across heat inputs. We used a paired Wilcoxon signed-rank test to compare trial-mean heating rates between thermocouple locations (back and surface) within clutches and to compare trial-mean heating rates between clutches and chambers. We used a Wilcoxon rank-sum test to compare trial-mean chamber heating rates between hydration treatments (wet and dry). To compare trial duration, clutch-mean heating rate, proportion hatched, proportional egg-volume loss and embryo mortality across hydration and heat input treatments, we created a composite variable “category” for the four experimental groups: Wet-High (WH), Wet-Low (WL), Dry-High (DH), and Dry-Low (DL). We used Kruskal–Wallis tests to compare these response variables across the 4 experimental groups then, if there were statistical differences, we applied Wilcoxon post-hoc tests to make comparisons among categories. To assess the egg volume loss, we calculated the mean initial and final egg volume for each clutch and used it to determine the mean proportion of volume lost during the trial. Non-parametric tests (Mann–Whitney–Wilcoxon, Kruskal–Wallis, and post hoc pairwise Wilcoxon tests) were performed using the “wilcox.test,” “kruskal.test,” and “pairwise.wilcox.test” functions, respectively, from the base R stats package (version 4.4.0), applying Holm correction for multiple comparisons.

To analyze 5-min heating rates, we fitted a generalized additive model (GAM) using the “gam” function from the “mgcv” package (Wood 2020). We constructed comparative models to test the effects of time as a continuous variable, along with thermocouple location (back and surface), heat input (high and low), and hydration (wet and dry) as two-level factors, with or without interactions. To assess effects of hydration and heat input, with or without interactions, on VT_Max_ we fitted comparative Generalized Linear Mixed Models (GLMMs) using the “glmmTMB” package (Brooks et al. 2017). Based on the Akaike Information Criterion (AIC), we used a Gaussian model with a logarithmic link function, which best fit the non-normal VT_Max_ data (vs. Gamma, Poisson, and Tweedie distribution families), improving variance stability and model interpretability (Brooks et al. 2017). In both 5-min heating rate and VT_Max_ models, we considered interaction terms up to the 3- or 4-way level in the candidate model set, as our aim was to evaluate how multiple environmental factors (e.g., hydration, heat input, location, and time) might interact. These interactions were included to test potential synergistic effects across factors. However, we only retained and interpreted interaction terms that were both statistically and biologically meaningful. In addition, we included sibship (clutch) identity as a random factor to account for multiple measurements within each trial, as recommended for hierarchical data structures (Zuur et al. 2009). We identified the best explanatory model according to the AIC (Akaike 1973; Bozdogan 2000), selecting the one with the lowest AIC value (Wang and Qun 2006). If 2 or more models had similar AIC values (i.e., differed by less than 2 units) we used the “aictab” function in the “AICcmodavg” package to calculate AIC weights and chose the model with the highest weight, closest to 1, as the best model (Symonds and Moussalli 2011). All analyses were performed and plotted in R V.4.4.0 (R Core Team 2025).

## Results

### Egg sizes, trial durations, and volume loss

Wet eggs were initially 1.9 times larger than dry ones, by volume (Wilcoxon rank sum test: *W* = 9813.5, *P* < 0.001; Table 1). Trial duration varied across our four treatment categories (Kruskal–Wallis test: *X^2^* = 18.036, *df* = 3, *P* < 0.001; Table 1). They did not differ between wet and dry clutches matched for heat input (Wilcoxon post-hoc tests: DL vs. WL and DH vs. WH, both *P* = 1.000). Regardless of hydration, trials were shorter under high heat input compared to low heat input, by about 40% (Wilcoxon post-hoc tests: WH vs. WL and DL, and DH vs. WL and DL, all *P* < 0.05; Table 1). Although initially wet eggs lost more volume than dry eggs, the proportional volume loss (15%) was similar across all 4 groups (Kruskal–Wallis test: *X^2^* = 1.987, *df* = 3, *P* = 0.575; Table 1).

**Table 1.** Summary descriptive data for wet and dry *A. callidryas* clutches, and the embryos therein, exposed to warming trials with high and low heat inputs.

	Hydration	Heat input	Location	Time-point	Mean ± SD	Range	*N*
Egg diameter (mm)	Wet			Initial	5.81 ± 0.80	3.99 − 7.55	276
	Dry			Initial	4.70 ± 0.52	3.15 − 5.83	280
Egg volume (µL)	Wet			Initial	108.45 ± 43.23	33.28 − 225.07	276
				Final	89.56 ± 35.79	20.49 − 177.75	160
	Dry			Initial	56.32 ± 17.88	16.40 − 103.65	280
				Final	47.77 ± 16.26	14.78 − 108.74	201
Proportional egg-volume loss (µL)	Wet	High			14.15 ± 4.61	10.57 − 22.79	7
		Low			16.00 ± 6.09	7.75 − 25.39	7
	Dry	High			13.76 ± 7.50	6.24 − 25.23	7
		Low			16.35 ± 5.62	11.07 − 27.43	7
Duration of trials (min)	Wet	High			67.14 ± 8.99	58 − 84	7
		Low			114.00 ± 16.53	87 − 139	7
	Dry	High			71.14 ± 19.32	50 − 108	7
		Low			112.86 ± 14.77	98 − 139	7
Proportion hatched	Wet	High			0.48 ± 0.30	0.12 − 0.88	7
		Low			0.51 ± 0.30	0.20 − 1.00	7
	Dry	High			0.51 ± 0.29	0.08 − 0.83	7
		Low			0.51 ± 0.28	0.22 − 1.00	7
Trial-mean water heating rate (°C/min)		High			0.39 ± 0.03	0.29 − 0.43	14
		Low			0.21 ± 0.01	0.18 − 0.22	14
Trial-mean clutch heating rate (°C/min)			Back		0.17 ± 0.05	0.10 − 0.27	28
			Surface		0.15 ± 0.04	0.09 − 0.25	28
	Wet	High			0.20 ± 0.02	0.17 − 0.24	7
		Low			0.12 ± 0.02	0.11 − 0.16	7
	Dry	High			0.21 ± 0.04	0.14 − 0.26	7
		Low			0.12 ± 0.01	0.10 − 0.13	7
Thermal gradient (at 30 min)	Wet	High			1.08 ± 0.63	0.07 − 1.95	7
		Low			0.84 ± 0.47	0.22 − 1.51	7
	Dry	High			0.94 ± 0.27	0.51 − 1.80	7
		Low			0.49 ± 0.27	0.21 − 1.03	7
Thermal gradient (at 60 min)	Wet	High			2.29 ± 1.16	0.78 − 3.90	6
		Low			1.04 ± 0.73	0.11 − 1.97	7
	Dry	High			1.79 ± 1.31	0.38 − 3.65	6
		Low			0.59 ± 0.28	0.17 − 0.96	7
Thermal gradient (at 90 min)	Wet	Low			1.43 ± 0.58	0.56 − 2.13	7
	Dry	Low			1.18 ± 0.50	0.43 − 1.85	7
Embryo VTMax (°C) by treatment	Wet	High			38.70 ± 0.96	36.55 − 42.32	111
		Low			38.34 ± 1.31	34.50 − 41.21	143
	Dry	High			37.62 ± 1.35	31.54 − 40.37	171
		Low			36.87 ± 2.31	29.27 − 40.03	168

### Heating rates

Our experimental setup successfully generated non-overlapping differences in water heating rate with heat input (Wilcoxon rank sum test: *W* = 196, *P* < 0.001; Table 1). Water heated about 80% faster under high heat input compared to low (*W* = 196, *P* < 0.001; Table 1). The air in the chamber warmed at a similar rate between hydration treatments (Wilcoxon rank sum test: *W* = 113, *P* = 0.503; mean ± SD, Wet: 0.15 ± 0.05°C/min, Dry: 0.16 ± 0.06°C/min, both *N* = 14). Clutch-mean heating rates were similar to their paired trial-mean chamber heating rates (Wilcoxon signed-rank test: *V* = 80.5, *P* = 0.368). Within clutches, trial-mean heating rates were 11.2% (0.019 °C/min) higher at the back than at the surface (Wilcoxon signed-rank test: *V* = 396, *P* = 0.001; Table 1). Clutch-mean heating rates varied across our four treatment categories (Kruskal–Wallis test: *X^2^* = 20.257, *df* = 3, *P* < 0.001; Table 1). They did not differ between wet and dry clutches matched for heat input (Wilcoxon post-hoc tests: DL vs. WL and DH vs. WH, both *P* = 0.958). However, regardless of hydration, clutches warmed about 70% faster under high vs. low heat input (Wilcoxon post-hoc tests: WH vs. WL and DL and DH vs. WL and DL, all *P* = 0.012; Table 1). Egg clutches warmed more slowly than the water behind them, and their mean heating rates overlapped slightly across heat input categories (Table 1). We removed data from 2 clutches with intermediate heating rates to generate a reduced data set with no overlap in clutch heating rates across heat input categories; all results were similar to those based on the full data set, which we present here.

**Fig. 2.**
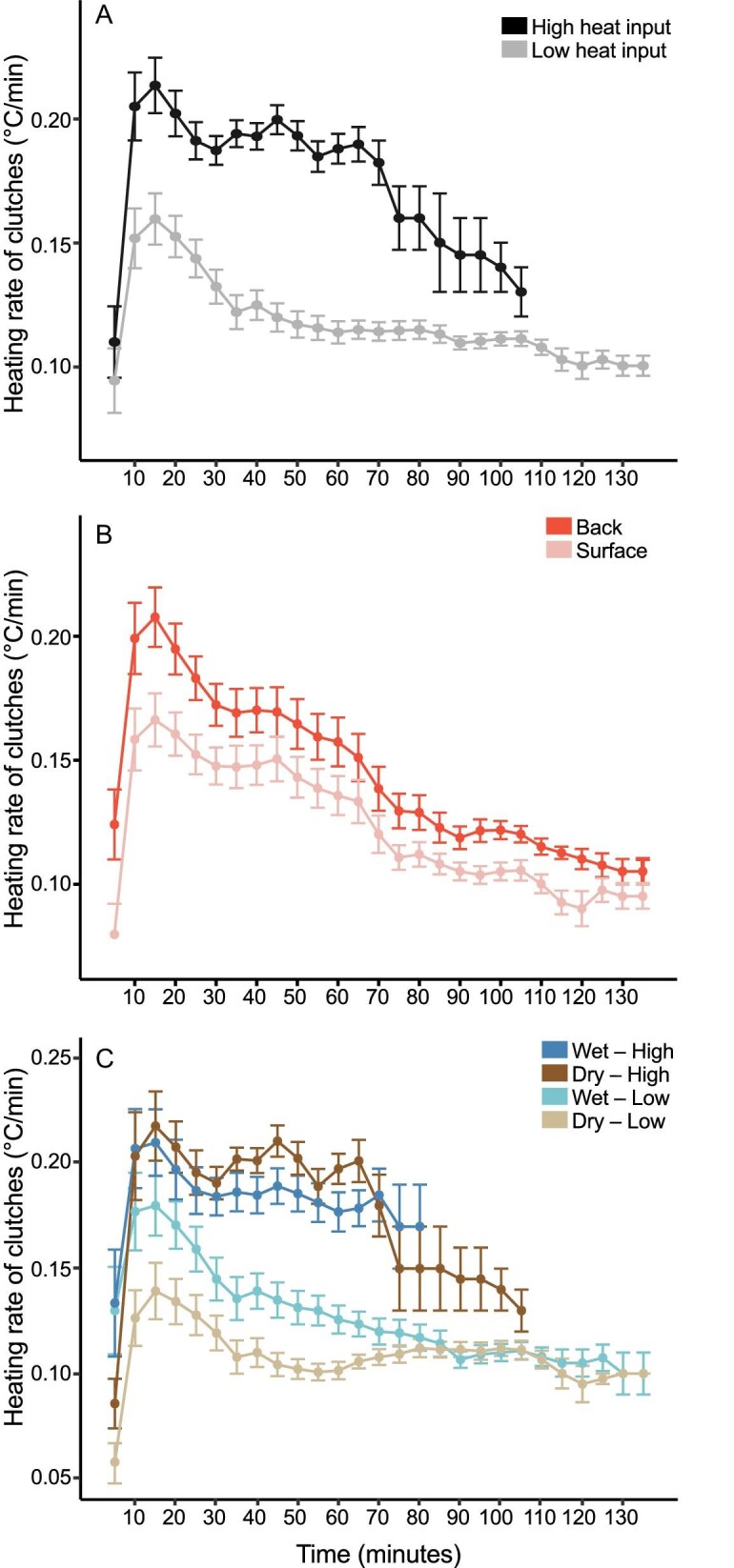
Temporal patterns in warming of *A. callidryas* clutches across heat inputs, thermocouple locations, and hydration × heat input categories, based on the heating rates calculated across sequential 5-min periods. (A) Heating rates of clutches exposed to high and low heat input over time. (B) Heating rates of clutches at back and surface thermocouple locations over time. (C) Temporal pattern of heating rates of wet and dry clutches exposed to high and low heat input. For all panels, points represent means across clutches and error bars represent SE. *N* = 28 clutches, 7 per treatment combination.

For 5-min heating rates of clutches, the best explanatory model (AIC weight = 1), showed significant main effects of thermocouple location, hydration and time, as well as 2-way interactions of location, hydration, and heat input with time, and a hydration × heat input interaction. There were no significant three or four-way interactions (Table 2). The heat × time interaction indicates differential heating rates of the clutches between high and low heat input over time (Fig. 2A; Tables 1 and 2). Heating rates increased rapidly at the start of trials for both heat inputs, then decreased and stabilized, and later decreased again, but were always higher with high heat input (Fig. 2A). The location × time interaction indicates that heating rates at each location varied over time; heating was consistently faster at the back than surface, generating thermal gradients within clutches that increased over time (Fig. 2B; Tables 1 and 2). Examining the main and interaction effects of hydration, that is, hydration × heat input and hydration × time, reveals a more complex pattern (Fig. 2C). At low heat inputs, the heating rates were initially higher for wet clutches, then after 70 min their heating rates converged. At high heat inputs, clutch heating rates were initially similar across hydration levels, then slightly higher in dry clutches, and lastly somewhat lower in the longest trials (Fig. 2C); with high heat input, all but one trial ended by 85 min. Overall, although heating rates varied among hydration × heat categories over time, heating rates were higher with high heat input, regardless of clutch hydration (Fig. 2C).

**Table 2.** Best-fit model to explain the heating rates of clutches over time.

	Estimate	Std. error	*t*-value	*P*-value
**5-min heating rates of clutches:**				
Intercept	0.184	0.010	17.712	**<0.0001**
Location (surface)	−0.038	0.007	−5.327	**<0.0001**
Hydration (drying)	−0.062	0.015	−4.230	**<0.0001**
Heat input (high)	0.027	0.015	1.760	0.078
Time	−0.001	0.000	−9.197	**<0.0001**
Location * Hydration	0.017	0.010	1.664	0.096
Location * Heat	−0.017	0.012	−1.415	0.157
Hydration * Heat	0.046	0.022	2.107	**0.035**
Location * Time	0.000	0.000	2.277	**0.023**
Hydration * Time	0.001	0.000	5.767	**<0.0001**
Heat * Time	0.001	0.000	2.961	**0.003**
Location * Hydration * Heat	0.005	0.017	0.308	0.758
Location * Hydration * Time	0.000	0.000	−0.746	0.456
Location * Heat * Time	0.000	0.000	0.704	0.481
Hydration * Heat * Time	0.000	0.000	−0.479	0.632
Location * Hydration * Heat * Time	0.000	0.000	−0.554	0.580

The sibship was included as a random factor. Bold values indicate statistically significant effects.

### Embryo thermal tolerance

**Fig. 3.**
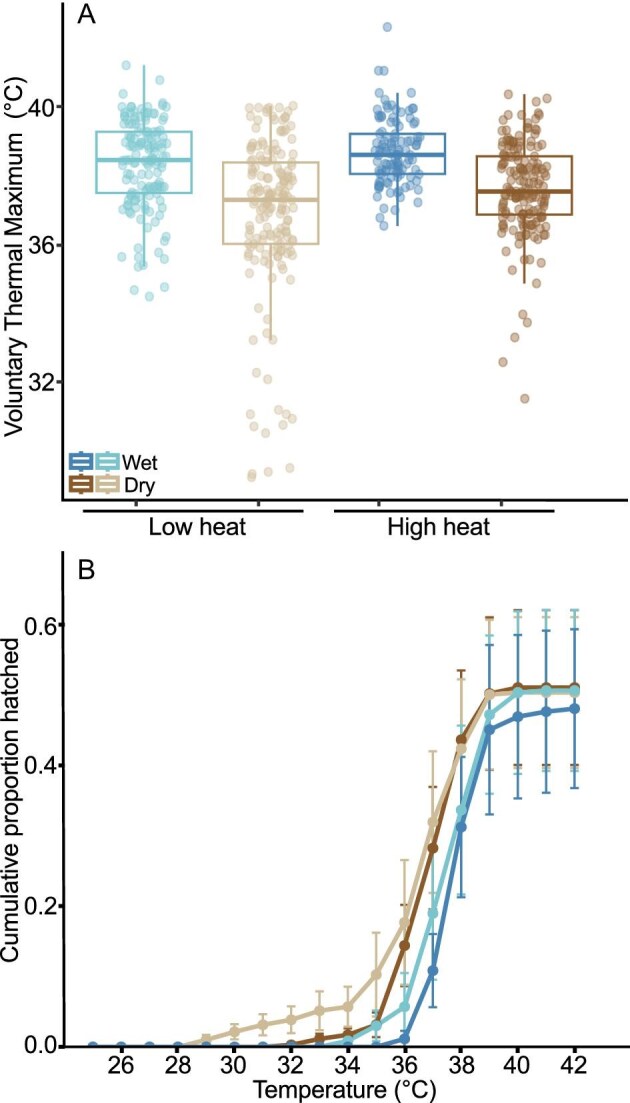
(A) Hatching temperatures (VT_Max_) of *A. callidryas* embryos in clutches at 2 hydration levels, (wet and dry) exposed to 2 heat inputs (high in dark, low in light colors). Each point represents an individual embryo, with data aggregated across 7 clutches per treatment and jittered horizontally for clarity. N embryos hatched per treatment: Wet–High = 111; Wet–Low = 143; Dry–High = 171; Dry–Low = 168. Box plots show the median and interquartile range, and whiskers show the 5th and 95th percentiles of the data. (B) Cumulative proportion of embryos hatched per clutch as temperature increased, with hydration × heat input category color coded as in (A). Lines represent curves fitted to clutch means (*N* = 7 clutches per treatment), and error bars indicate standard error across clutches.

For VT_Max_, the best explanatory model (AIC weight = 1) showed a significant main effect of hydration, no effect of heat input, and no hydration × heat interaction (Fig. 3; Table 3). Across heat input levels, embryos in dry clutches hatched at temperatures 1.3°C lower than those in wet clutches (Fig. 3A; Tables 1 and 3). The hydration effect was also evident in the cumulative proportion hatched over temperature, which revealed a clear sequential pattern: hatching began at the lowest temperatures in dry clutches under low heat input, followed by dry clutches under high heat, and finally, wet clutches under low and high heat input (Fig. 3B).

**Table 3.** Best-fit model to explain the effects of hydration and heat input on embryo VT_Max_.

	Estimate	Std. error	*z*-value	*P*-value
Voluntary thermal maximum:				
Intercept	3.651	0.007	513	**<0.0001**
Hydration (drying)	−0.036	0.010	−3.60	**<0.0001**
Heat input (high)	0.007	0.011	0.70	0.519
Hydration * Heat	0.009	0.015	1.00	0.525

The sibship was included as a random factor. Bold values indicate statistically significant effects.

All embryos that hatched survived successfully for 5 days after heating and were released to the Experimental Pond. Some embryos did not hatch during heating; for these, we induced hatching with mechanosensory stimulation, and all survived for 5 days after heating. Embryo deaths occurred during six of the 28 experiments: 8 individuals each in WL and DL, 6 in WH and none in DH. Proportional mortality did not differ among hydration × heat input categories (Kruskal–Wallis test: *X^2^* = 3.594, *df* = 3, *P* = 0.308). Very little hatching occurred at temperatures over 40°C (just 2.9%) and only 3 individuals hatched at temperatures above 41°C (0.7% of hatched embryos, at 41.1, 41.2, and 42.3°C, all from the WH group). There was no difference in the proportion of embryos hatched between categories (one-way analysis of variance (ANOVA): *F*_3,24 _= 0.016, *P* = 0.997; Table 1). Overall, 49.80 ± 0.02% of embryos remained unhatched at the end of trials.

## Discussion

### Methodological challenges in assessing the thermal tolerance of terrestrial frog embryos

Studying thermal tolerance in terrestrial frog embryos is crucial due to their unavoidable exposure to high temperatures and desiccation risks during development. Methods for assessing ectotherms' thermal tolerance at different life stages often face limitations in controlling variables and accounting for species-specific responses, complicating the observation of consistent patterns (Terblanche et al. 2007; Moyano et al. 2017; Jørgensen et al. 2021; Desforges et al. 2023). Although previous studies have used CT_Max_ and LT_50_ to assess thermal tolerance in anuran embryos (Turriago et al. 2015), the first VT_Max_ measurements highlighted the difficulty of controlling warming rates without also controlling evaporation (see Fig. 3 in Guevara-Molina et al. 2022). This confounded variation meant that a negative effect of warming rate on embryo VT_Max_ could overshadow the effect of drying, leaving the actual modulators of embryo responses unresolved (Guevara-Molina et al. 2022). To advance studies on the thermal ecology of frog embryos, it was essential to develop a method that not only provided better control over egg-warming rates but also minimized evaporation during experiments to maintain the initial hydration state of clutches. By adjusting the heating rates and minimizing heat loss to evaporation, we were able to explore the separate and combined effects of heating rate and hydration on embryos’ hatching decisions.

### Methodological advances clarify the effect of hydration on embryo VT_Max_

Our new experimental method successfully addressed the wide difference in mean heating rate and trial duration evident in Guevara-Molina et al. (2022), achieving more consistent conditions across hydration treatments. This methodological refinement equalized heating rates and trial durations while substantially reducing changes in egg volume during trials, especially for hydrated clutches. Using this improved approach provided clearer insights into the role of hydration in embryo thermal tolerance. Notably, lower hatching temperatures of embryos in dry clutches cannot be simply a byproduct of faster warming, due to their limited capacity for evaporative cooling. Our findings demonstrate that dehydration reduces thermal tolerance without interacting effects of heat input, suggesting that drying itself plays a critical role in lowering hatching temperatures. Although drying appeared to reduce hatching temperature slightly more under slower warming (Fig. 3A–B; Table 1), this difference was not statistically robust enough to indicate a main or interaction effect of heat input. These findings are consistent with those of Guevara-Molina et al. (2022) who also found that drying reduced VT_Max_ of embryos in small groups, removed from their clutch, jelly, and leaf, but there was no effect of warming rate in that context. While the size of the drying effect varies—from 1.3°C in this study to 4.7°C in isolated egg-groups under free evaporation (Guevara-Molina et al. 2022)—drying consistently reduces behavioral thermal tolerance.

In contrast, the effect of warming rate on VT_Max_ was inconsistent. By substantially reducing water loss, we improved control over hydration during heating. Under our new protocol, the mean egg-volume loss was reduced and equalized to 15% of initial volume (19 µL in wet clutches and 9 µL in dry clutches), compared to 53% of initial volume (76 µL) for wet clutches and 41% (17 µL) for dry ones under free evaporation conditions (Guevara-Molina et al. 2022). With evaporation reduced, there was no clear or significant effect of warming rate on VT_Max_. If anything, embryos in dry clutches showed slightly higher VT_Max_ with faster warming—opposite to the reduced VT_Max_ measured for faster-warming embryos in dry clutches under our earlier free-evaporation protocol (Guevara-Molina et al. 2022).

### Advancing the understanding of behavioral thermal tolerance in terrestrial frog embryos

The effects of warming rates on physiological thermal tolerance (CT_Max_) have shown considerable variation across studies (e.g., anurans: Anderson and Andrade 2017; Agudelo-Cantero and Navas 2019; Guevara-Molina et al. 2020; Turriago et al. 2023; other ectotherms: Vinagre et al. 2015; Moyano et al. 2017; Penman et al. 2023). This underscores the complexity of interpreting thermal tolerance data and highlights the species-specific nature of responses, emphasizing the need to consider heating protocols, ecological context, and physiological factors for accurate assessments (Ruthsatz et al. 2024). Unlike CT_Max_, much less is known about how warming rates influence VT_Max_ across different contexts. Behavioral limits such as VT_Max_ are typically reached before physiological ones during heating, reflecting individual decisions to avoid overheating rather than a physiological failure (Camacho and Rusch 2017; Guevara-Molina et al. 2020). These decisions are influenced by a variety of environmental and intrinsic factors, including hydration status and previous thermal exposure. In anurans, dehydration consistently reduces VT_Max_ in embryos and juveniles (Guevara-Molina et al. 2020; Guevara-Molina et al. 2022). In hydrated juvenile bullfrogs, higher warming rates were positively related to CT_Max_, but did not correlate with VT_Max_ (Guevara-Molina et al. 2020), suggesting these thresholds may be driven by different mechanisms. Similarly, in embryos, warming rates were confounded with hydration effects on VT_Max_ (Guevara-Molina et al. 2022).

By manipulating heat input and controlling evaporation to generate variation in warming rate independent of hydration, we clarified the consistent direct effect of dehydration in reducing VT_Max_. Despite controlling evaporation, the method revealed complexity in the thermal dynamics of egg clutches, including thermal gradients that varied among clutches and over time. We estimated the VT_Max_ based on temperature at the nearest thermocouple while acknowledging that, given the gradients, this does not precisely reflect individual temperature for all eggs, that is, some individual values may be slightly over- or underestimated. As this creates some noise in the data it may reduce, but should not increase, our chance of detecting treatment effects. Since, unlike in Guevara-Molina et al. (2022), we effectively generated clutch-mean heating rates that were similar across hydrations but differed across heat inputs, we do not attempt to estimate warming rates for individual eggs. We cannot entirely rule out an effect of warming rate on embryo thermal tolerance. However, if such an effect exists, detecting it would require either larger differences in warming rate or more precise measurements of individual egg temperatures—for example, using thermal cameras or simplified setups with individual embryos. Embryo-specific warming rate measurements could refine our understanding of the interactive effects of hydration, heat input, and warming rate, offering deeper insights into the modulation of thermal tolerance. The integration of behavioral thermal tolerance with hydration, warming rates, and evaporative water loss underscores the challenges faced by ectotherms in balancing hydration and thermal stress management (Guevara-Molina et al. 2020; Guevara-Molina et al. 2022).

The mechanisms underlying a behavioral escape-response to heat, and its modulation by other factors, include the risk assessment process as much as variation in physiological tolerance. For terrestrial anuran embryos whose survival depends on hydration, behavioral rules are shaped by the need to avoid both overheating and excessive water loss. These risks are related in multiple ways. Well-hydrated amphibians, including terrestrial egg clutches, have substantial capacity for evaporative cooling, which increases their thermal safety margin (Sunday et al. 2014; Guevara-Molina et al. 2022). Dehydration reduces this capacity and—under natural free-evaporation conditions—accelerates warming, forcing these animals to rely on behavior to avoid critical overheating (Sunday et al. 2014). The escape-hatching response of these embryos to drying indicates an ability to sense dehydration independent of warming (Salica et al. 2017), and dry conditions also represent a direct physiological risk. Dehydration affects cellular function and increases metabolic costs, as the rate of cellular damage exceeds the rate of repair (Rezende et al. 2011), and systemic effects, such as increased blood viscosity and cardiac effort impose additional costs (Hillman 2018). Prolonged exposure to heat can intensify these impacts, even at lower heating rates (e.g., Peck et al. 2009; Rezende et al. 2011). In general, embryos might expect warming cues to be accompanied by drying cues, and their escape-hatching behavior suggests they have evolved to respond to the combined risk of thermal and hydric stress. Reduced thermal tolerance under dehydration may reflect an adaptive response to these synergistic threats. Future studies should investigate the sensory mechanisms by which embryos perceive temperature and hydration, and how they process both current conditions at the rate at which these cues change.

### Linking embryo behavior to heat-induced hatching: Potential underlying mechanisms

During warming experiments, we observed that embryos exhibit behavioral changes *in ovo*, suggesting they perceive increasing temperatures well before reaching and expressing their VT_Max_ (see Supplementary Fig. S1 and Videos). We first observed increased movement (position changes), then expansion of the external gills toward the egg surface, a darkening of embryo coloration (melanophore expansion) and, sometimes, arrhythmic shaking movements as the last behavior prior to hatching, which is associated with directed shaking behavior (Cohen et al. 2016; Warkentin et al. 2017; Güell et al. 2022; Jung et al. 2022). Some of these changes have been observed in fish embryos, which increased their movements *in ovo* when they were 2–3°C away from reaching their CT_Max_ (Cowan et al. 2023). *Agalychnis callidryas* embryos orient in oxygen gradients within their egg and increase their rate of position changes under hypoxia, interpreted as searching for the high-oxygen zone, prior to hypoxia-cued hatching (Rogge and Warkentin 2008; Warkentin et al. 2017). We found substantial thermal gradients within clutches, suggesting embryos’ increased movement rate under warming may, similarly, represent searching for a thermally better position. Turtle embryos, for example, move within eggs to optimize their position in thermal gradients, mediated by TRPA1 and TRPV1 thermal receptor sensors (Ye et al. 2021), which form the molecular basis of embryonic behavioral thermoregulation. Similar mechanisms may help other embryonic ectotherms sense temperature and regulate hatching. Additionally, warmer water holds less oxygen, and both increased activity and increased metabolism at higher temperatures will increase oxygen demand. Thus, oxygen stress may contribute to heat-induced hatching. In addition, as eggs lose water, the toxic waste product ammonia becomes more concentrated (Méndez-Narvaez and Warkentin 2022). Ammonia toxicity has been documented in frog embryos and larvae (e.g., Daam et al. 2020; Méndez-Narvaez and Warkentin 2022). An elevated ammonia level induces hatching even in well-hydrated eggs and may serve as a cue indicating egg drying (Lisondro-Arosemena et al. 2024). Ammonia would increase in concentration at higher temperatures, likely due to elevated metabolic rates (e.g., Chew et al. 2005; Barbieri and Bondioli 2015; Kir et al. 2019). Consequently, at higher temperatures, toxic concentrations could be reached more quickly, accompanied by dehydration. This could lead to a lower thermal tolerance as a byproduct. The arrhythmic shaking we observed suggests possible neurological impacts on motor skills, and we observed reduced hatching at the high end of tested temperature range, even with many embryos still alive. This suggests either impaired hatching ability below lethal thresholds or variability in decision rules. Overall, the observed pre-hatching behaviors suggest that thermal tolerance is shaped by multimodal risk perception when drying and warming are combined.

## Conclusions

Our study advances research in the thermal ecology of terrestrial amphibian embryos and reveals the complexity of thermal ecology in frogs at early life stages. Using a novel heating method, we supported a consistently negative effect of drying on thermal tolerance, independent of heating rate. This effect varied in magnitude across studies and contexts, suggesting that embryo responses involve complex risk assessment mechanisms. While our study clarifies the importance of dehydration in modulating embryonic thermal tolerance, it also reveals thermal complexity within clutches. This should be assessed under more natural conditions, which will require further refinements in measurement techniques. Then, individual-level manipulations in the laboratory can be better tailored to match conditions in nature. Overall, we recommend that key variables such as hydration states, dehydration rate, and warming rates should be carefully considered to better understand how tropical amphibian species with terrestrial oviposition respond to thermal and water stress during embryo development. Integrating these factors into future research will provide a more comprehensive understanding of the complex interactions that shape embryonic thermal tolerance and behavioral responses under changing environmental conditions. Given the structural diversity and varied oviposition sites of amphibians’ terrestrial egg clutches, we suggest that comparative studies across species are crucial to fully understand how these factors may shape embryo responses to environmental climate stress. Future research should aim to identify the physiological and behavioral mechanisms driving the observed patterns and investigate the role of evaporative cooling in regulating clutch thermal and water dynamics under natural conditions.

## Supplementary Material

obaf023_Supplemental_Files

## Data Availability

Analyses reported in this article can be reproduced using the data, R code, and videos provided in the supplementary materials.
