# Supplementary figures and images for: Heat-Induced Hatching: Clarifying Effects of Hydration and Heating Rate on Behavioral Thermal Tolerance of Red-Eyed Treefrog Embryos
Source: Integr Org Biol. 2025 Jun 5;7(1):obaf023. doi: 10.1093/iob/obaf023 (PMC12257936; doi:10.1093/iob/obaf023)

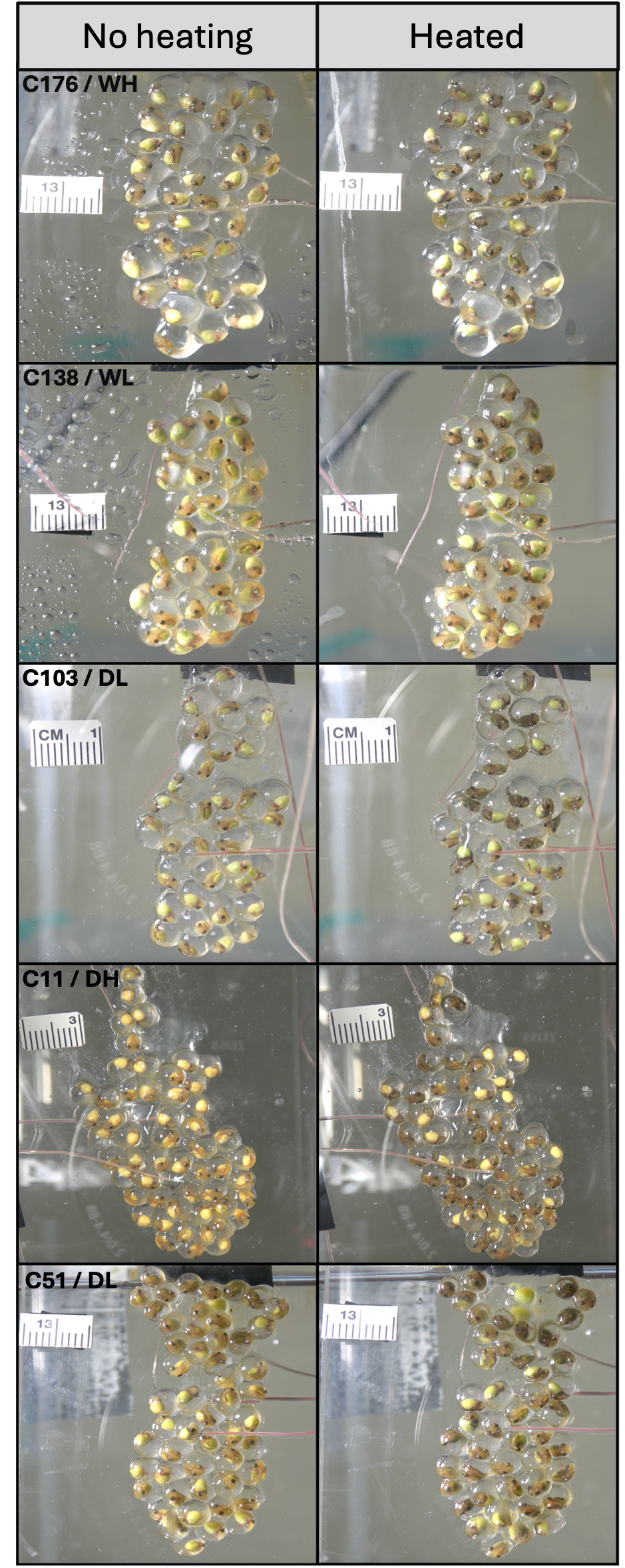

Supplement: obaf023_Supplemental_Files [file obaf023_supplemental_files.zip › Supplementar figure 1.png]
